# Iodine Nutritional Status of School Children in Nauru 2015

**DOI:** 10.3390/nu8090520

**Published:** 2016-08-23

**Authors:** Chun-Jui Huang, Chi-Lung Tseng, Harn-Shen Chen, Chanda Garabwan, Samuela Korovo, Kam-Tsun Tang, Justin Ging-Shing Won, Chang-Hsun Hsieh, Fan-Fen Wang

**Affiliations:** 1 Division of Endocrinology and Metabolism, Department of Medicine, Taipei Veterans General Hospital, Taipei 11217, Taiwan; chunjui0501@yahoo.com.tw (C.J.-H.); chenhs@vghtpe.gov.tw (H.-S.C.); tang8228@gmail.com (K.-T.T.); gswon@vghtpe.gov.tw (J.G.-S.W.); 2 Faculty of Medicine, School of Medicine, National Yang-Ming University, Taipei 11217, Taiwan; chilong1223@yahoo.com.tw; 3 Institute of Public Health, National Yang-Ming University, Taipei 11217, Taiwan; 4 Division of Gastroenterology, Department of Medicine, Taipei Veterans General Hospital, Taipei 11217, Taiwan; 5 Division of Gastroenterology, Department of Internal Medicine, Taoyuan General Hospital, Ministry of Health and Welfare, Taoyuan 33004, Taiwan; 6 Institute of Clinical Medicine, National Yang-Ming University, Taipei 11217, Taiwan; 7 Nauru Public Health Center, Republic of Nauru; chanda.garabwan@health.gov.nr; 8 Republic of Nauru Hospital, Republic of Nauru; korovousam@gmail.com; 9 Division of Endocrinology and Metabolism, Department of Internal Medicine, Tri-Service General Hospital, National Defense Medical Center, Taipei 114, Taiwan; 10324@yahoo.com.tw; 10 Department of Medicine, Yangming Branch, Taipei City Hospital, Taipei 11146, Taiwan

**Keywords:** iodine, urinary iodine concentration, iodized salt, nutrition, Nauru

## Abstract

Little is known about iodine nutritional status in island countries in the Pacific Ocean. The primary objective of this study was to report for the first time the iodine nutritional status of people in Nauru. In addition, sources of iodine nutrition (i.e., water and salt) were investigated. A school-based cross-sectional survey of children aged 6–12 years was conducted in three primary schools of Nauru. Urinary iodine concentration (UIC) was determined by spot urine samples. Available water and salt samples in Nauru were collected for the measurement of iodine content. A food frequency questionnaire was conducted. The median UIC was 142 μg/L, and 25.2% and 7.4% of the population had median UIC below 100 μg/L and 50 μg/L, respectively. Natural iodine-containing foods such as seaweeds and agar were rare. Iodine was undetectable in Nauruan tank water, filtered tap water, and raindrops. Of the analyzed salt products, five kinds were non-iodized, and three were iodized (iodine content: 15 ppm, 65 ppm, and 68 ppm, respectively). The results indicate that the iodine status in Nauruan school children is adequate. Iodized salt may serve as an important source of iodine nutrition in Nauru.

## 1. Introduction

Iodine is an essential micronutrient required for the synthesis of thyroid hormone, which is crucial to the growth and development of human beings. The spectrum of iodine deficiency disorders (IDD) includes thyroid dysfunction with or without goiter, intellectual impairments, growth retardation, cretinism, increased pregnancy loss, and infant mortality [1]. According to the result of meta-analyses, children living in areas with iodine deficiency had an intelligence quotient of 6.5–12.45 points lower than the intelligence quotient of those living in iodine-sufficient areas [2,3]. Serious health consequences such as cretinism and severe brain injury can be the manifestations of severe iodine deficiency [4]. Even mild iodine deficiency in pregnancy may lead to poorer cognitive outcomes in children, thus impairing their learning capacity and affecting the social and economic development of their countries [5,6,7]. These consequences of iodine deficiency are preventable by appropriate iodine intervention [8]. Therefore, the World Health Organization (WHO), the United Nations Children’s Fund (UNICEF), and the International Council for the Control of Iodine Deficiency Disorders (ICCIDD) (now known as Iodine Global Network or IGN) put great efforts into eliminating IDD worldwide, and recommended universal salt iodization as the strategy [9]. During the past decade, the number of countries with adequate iodine nutrition has steadily increased from 67 to 112 in 2014 [10,11,12]. However, the iodine status of several island countries in the Pacific Ocean is unknown.

Iodine deficiency is traditionally believed to be more prevalent in inland regions and mountainous areas [1]. It has not been considered as a major public health issue in the Pacific islands, as it was believed that Islanders have ready access to iodine-rich seafood from the surrounding ocean. Seawater evaporates into the atmosphere and drops into the soil through rain, supporting crop growth in iodine-sufficient soils rich in iodine [1]. However, a recent survey performed in the island of Tanna in Vanuatu reported a high prevalence of iodine deficiency [13]. Another study in Samoa provided evidence of iodine deficiency in the Pacific region [14]. Emerging data worldwide revealed that IDDs are not confined to remote mountainous areas, but are a global nutritional problem that affects island nations as well [15].

Nauru is a 21 km^2^ oval-shaped island country in the Pacific Ocean, located 42 km south of the equator. As of 2015, Nauru had around 10,000 residents, those aged 18 years or younger accounting for about 50% of the population. Without river and water reservoirs, there are limited natural fresh water resources. Islanders rely on rooftop storage tanks collecting rainwater and a seawater desalination plant. Currently, there are no data on iodine nutrition in Nauru. The aim of the present study was to report for the first time the iodine status of people in Nauru. The sources of iodine in the diet (i.e., water and salt) were also investigated.

## 2. Materials and Methods

### 2.1. Study Design

**Figure 1:**
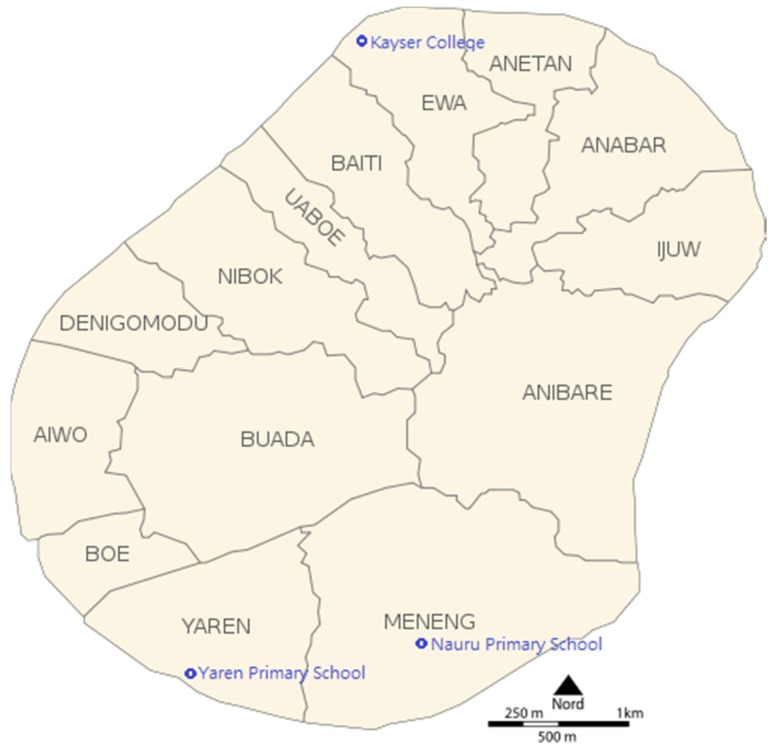
Map of Nauru with the location of the three primary schools.

This survey was a collaborative initiative between the Nauru Ministry of Health, Taiwan Ministry of Foreign Affairs, and Taipei Veterans General Hospital, Taiwan. Ethical approval was granted by the Nauru Non-communicable Disease Steering Committee, Nauru Ministry of Health (20150917). This research was designed as a school-based survey to collect nationally representative data of iodine status in Nauru. All three primary schools (Yaren Primary School, Nauru Primary School, and Kayser College, Figure 1) in Nauru with students aged 6–12 years participated in the study. Participating students were randomly selected by the Statistics Division of the Ministry of Education, Nauru, to represent their age, gender, and region of residence. Students were asked to provide a random spot urine sample and fill out a short Food Frequency Questionnaire (FFQ). Informed consent was signed by one of the parents of the enrolled school children. A total of 242 students (126 males, 116 females) aged 6–12 years successfully participated in this study. To determine the source of iodine nutrition, water samples from different regions were collected and analyzed. The iodine content in salt products in Nauru was also measured.

### 2.2. Urinary Iodine Analysis

Urine samples were collected in 2015. Each child was accompanied to the toilet by one of the medical staff members of Nauru Public Health Center or the teacher at the school to ensure adequate urine collection. After the child urinated into a plastic cup, aliquots of urine were immediately transferred to a 2-mL frozen tube. Urine samples were stored at 4 °C in Nauru Public Health Center before being transported to the Research Core Laboratory of Taipei City Hospital, Yangming Branch, Taiwan, where urinary iodine concentration (UIC) was measured.

UIC was assayed by a modified microplate method based on the Sandell-Kolthoff (S-K) reaction using ammonium persulfate as the oxidizing agent (details of the analysis methods can be obtained from the authors on request) [16,17,18,19,20]. Pooled urine samples with low, medium, and high iodine concentrations were included in each plate as internal controls to verify the accuracy of the assay. External quality control samples were provided by the Ensuring the Quality of Urinary Iodine Procedures (EQUIP) program and were tested three times a year to ensure the quality of UIC measurements. All samples were analyzed in duplicate for two runs. Samples with readings >350 μg/L were diluted with water to fit the calibration curve. The measurement was repeated for samples with discordant duplicate values exceeding 15%. Samples with readings <25 µg/L were measured by a modified low UIC protocol [14]. The intra- and inter-assay coefficients of variation were <10%.

### 2.3. Water Iodine Analysis

The 14 districts in Nauru (Figure 1) were grouped into three zones, according to the geographical location: southwestern region, zone 1 (Buada, Aiwo, Boe, and Yaren); northeastern region, zone 2 (Baitsi, Uaboe, Nibok, and Denic); and eastern region, zone 3 (Meneng, Anabar, Anibare, Anetan, Ewa, and Ijuw). Tank water in 13 different locations (four samples from zones 1 and 2 and five samples from zone 3) in Nauru was collected, and the water iodine content was determined using the same microplate method used to measure UIC. Iodine content in rain water and filtered tap water was also measured.

### 2.4. Salt Iodine Analysis

All the available salt products (eight types and four brands) in the market during the study period were collected. Salt iodine content was determined by the colorimetric titration method using sodium thiosulfate as the reagent [9]. In brief, 50 g of salt was dissolved in water to form a 250 mL solution and mixed with 1 mL of 2 N sulfuric acid. If potassium iodate was present in the salt product, the solution would turn yellow when 5 mL of 10% potassium iodide solution was added. The reaction mixture was titrated with 0.005 N sodium thiosulfate using starch (2 mL) as the indirect indicator. The concentration of iodine in salt was read from a precalculated table. The sensitivity was 1.7 mg/kg (ppm).

### 2.5. Food Frequency Questionnaire

The qualitative, non-validated FFQ consisted of eight questions, all in the form of “How many days in a week do you eat a certain kind of food?” The types of food included in the survey were (1) rice; (2) noodles; (3) bread; (4) meat; (5) vegetables; (6) fruits; (7) seaweeds; and (8) agar (jelly). Common foods were chosen from the major classes of nutrients (carbohydrate, protein, fibers, etc.) along with naturally iodine-rich seaweeds and agar. Participants were asked to tick the closest answer, whether it was 7 days, 5 days, 3 days, 1 day, or never. The questionnaire was written in English. The FFQ was administered individually to each student in the local language by a teacher or medical staff member.

### 2.6. Statistical Analysis

The iodine status was expressed as median UIC for the sampled population. The criteria for classifying the iodine nutrition of a population proposed by the WHO/UNICEF/IGN criteria are <20 μg/L as severe iodine deficiency; 20–49 μg/L as moderate iodine deficiency; 50–99 μg/L as mild iodine deficiency; 100–199 μg/L as adequate iodine nutrition; 200–299 μg/L as above the requirement; and ≥300 μg/L as excessive. In addition, not more than 50% of the population should have a UI < 100 μg/L, and not more than 20% of the population should have a UI < 50 μg/L [9].

Descriptive statistics and hypothesis testing were analyzed by the Statistical Package for the Social Sciences (SPSS) software, version 18.0. The significance of variation among the various statuses was evaluated by Kruskal–Wallis test. A two-tailed *p* value of less than 0.05 was considered as statistically significant.

## 3. Results

### 3.1. UIC Level

As shown in Table 1, the median UIC for the Nauruan population aged 6–12 years was 142 μg/L (95% CI: 132–154). Males and females had a similar median UIC, with 145 μg/L (95% CI: 131–159) in males and 139 μg/L (95% CI: 126–156) in females. The median UIC of different age groups ranged from 130 to 153 μg/L (131 to 152 μg/L in males and 126 to 170 μg/L in females). As shown in Table 2, 25.2%, 7.4%, and 3.1% of the population had UIC <100, <50, and <20 μg/L, respectively. There were no statistically significant differences between UIC among different age groups and gender.

As shown in Table 1, people living in different areas of Nauru had a median UIC ranging from 130 μg/L to 152 μg/L. The median UIC of males and females in the three regions were 131–154 μg/L and 129–148 μg/L, respectively. The overall percentage of males and females with UIC < 100 μg/L was less than 50%, and less than 20% of children surveyed had UIC < 50 μg/L across all regions. There were no statistically-significant differences between UIC in the different geographic areas.

### 3.2. Water Iodine Level

All the tank water, filtered tap water, and the raindrops collected from different areas had an iodine concentration level below the functional sensitivity level of the assay (<1.6 μg/L).

### 3.3. Salt Iodine Level

Of the eight types (four brands) of salt products, three were iodized and five were non-iodized. The three kinds of iodized salt had an iodine content of 15 ppm, 65 ppm, and 68 ppm, respectively. Iodine was undetectable in all five non-iodized salts.

### 3.4. Food Frequency Questionnaire

As shown in Table 3, the most common dish on the table of Nauruan diet was rice and meat. Overall, 88% of people ate rice and 61.4% of the people ate meat every day. The median days of bread intake were 5 (interquartile range: 3–7 days), and the median days of intake of noodles, fruits, and vegetables were 3. When the people were questioned regarding the consumption of natural iodine-containing foods such as seaweeds and agar, 87.6% and 72.3% of them reported that they hardly ever had access to these types of food.

## 4. Discussion

To our knowledge, this is the first time that the iodine status was surveyed and reported for a country in Micronesia in the Central Pacific. In this school-based cross-sectional survey of school children aged 6–12 years in Nauru, we found that the median UIC was 142 μg/L, and 25.2% and 7.4% of population had median UIC below 100 μg/L and 50 μg/L, respectively. According to the WHO/UNICEF/IGN criteria, Nauru is an iodine-sufficient country [9]. There were no statistically significant differences in the median UIC among the different age groups, gender, or regions of Nauru.

The current study demonstrated sufficient iodine nutrition in the island country of Nauru, but the source of iodine was not related to water. In the absence of a river or water reservoir in Nauru, water supply primarily comes from tank water containing rainwater collected directly from the rooftops of Nauruan households or filtered tap water produced by sea water desalination, in which iodine content was undetectable. Iodine was also not measurable in the rain water collected in Nauru.

According to the results of the short FFQ in this study, natural iodine-containing foods such as seaweeds and agar are rarely consumed by children in Nauru. When the Nauruan diet relied more on fresh fish and meat in the 1970–1980s, the surrounding ocean may have served as the source of iodine-containing food. However, globalization and World Trade Organization agreements caused the Pacific island countries to undergo a period of nutrition transition [21,22]. As a result, the modern diet demanded more packaged imported foods, processed meats, and refined starch and oil [23]. Along with the decreased consumption of locally produced plants and animals, vulnerability to iodine deficiency increased. This phenomenon is observed not only in Nauru but also in several Pacific island countries, which makes iodine surveys extremely important [21,22]. 

Nauru holds a voluntary salt iodization policy. However, the percentage of households using iodized salt was not included in this study. Because Nauruan people do not eat bread every day, mandatory fortification of bread with iodized salt as done by some nearby countries may not be a feasible option to improve iodine intake [24,25,26]. If deterioration of iodine nutrition occurs, increasing the percentage of households using iodized salt is the most effective strategy to eliminate iodine deficiency, such as a mandatory salt iodization program.

In the present study, the median UIC of 142 μg/L does not necessarily mean sufficient iodine nutrition for pregnant women [27]. The criterion for iodine sufficiency is much higher in pregnancy (UIC 150–249 μg/L) [9]. This is because renal clearance of iodine is increased during gestation, and the fetus depends on maternal iodine for the synthesis of thyroid hormone [5]. Therefore, the recommended daily iodine intake in pregnancy is 250 μg, which is higher than the recommended amount of 150 μg for adults [9]. Thyroid hormones are essential for neuron formation, migration, and differentiation, thus playing a key role in fetal brain development [4,28]. Epidemiological evidence suggests that even mild iodine deficiency in pregnancy may result in poorer cognitive outcomes in childhood [5,7]. Pregnant and lactating women have long been considered the most vulnerable population to iodine deficiency [4,5,6,7]. Unfortunately, women of childbearing age are often unaware of their pregnancy during the first 2 months of gestation. This warrants an iodine nutrition survey in pregnant, breastfeeding women and those of childbearing age. 

There are several limitations to this study. First, the FFQ is a simple questionnaire which did not include key dietary sources of iodine such as salt and seafood. With very few local fishermen on the island, fresh fish is extremely expensive and seldom appears on the dining table. Dairy products were also not common. Children were confused about the differences between fish, meat, pork, and seafood, as well as the meaning of dairy products. Therefore, intake of fish and dairy products were omitted in the questionnaire. Second, sources of dietary salt and household coverage of iodized salt were not evaluated in this study. While promoting iodized salt as the source of iodine nutrition may be feasible, the risk of worsening hypertension should be taken into consideration in a community where processed foods are popular and salt consumption is likely to be increasing. Further research investigating trends in salt consumption and sources of dietary salt is warranted. According to the WHO/UNICEF/IGN, the school-based survey using spot urine samples is a recommended method to assess iodine status in the general population [9]. However, it is possible that the nutrition condition in the households may be different from that in schools. Including household coverage of iodized salt in future studies is recommended. In addition, the current median UIC level (142 μg/L) suggesting sufficient iodine nutrition in the general population cannot be applied to pregnant women. A survey of iodine status in pregnant and lactating women is needed.

## 5. Conclusions

We conclude that iodine nutritional status is sufficient in Nauru, based on spot urine samples collected in 2015. There were no significant variations in UIC among the different studied age groups and regions in Nauru. Iodized salt may serve as an important source of iodine nutrition in Nauru. The risk of iodine deficiency in vulnerable subgroups may be overlooked when relying on the UIC level of school children to determine the country’s iodine status [29]. More research is warranted to assess the iodine nutritional status in different subgroups, such as pregnant women, lactating women, and neonates. Reassessment of iodine status is recommended every 3–5 years and whenever national iodization policy changes.

## Figures and Tables

**Table 1:** Urinary iodine levels (μg/L) by age, gender, and geographic area.

	Total (*n* = 242)		Males (*n* = 126)		Females (*n* = 116)	
	Median	95% CI	Median	95% CI	Median	95% CI
**Age**						
6–12 years	142	132–154	145	131–159	139	126–156
6–8 years (*n* = 79)	146	134–167	131	99–155	148	112–175
8–10 years (*n* = 63)	153	130–168	134	106–162	170	129–208
10–12 years (*n* = 100)	130	120–155	152	119–183	126	114–151
**Region**						
West-southern (*n* = 71)	150	131–170	150	131–177	148	125–170
West-northern (*n* = 76)	130	110–147	131	99–155	129	109–181
Eastern area (*n* = 95)	152	131–167	154	126–169	141	121–168

**Table 2:** Distribution of urinary iodine levels <100 μg/L, <50 μg/L, and <20 μg/L by age and gender.

Age (Years)	6–12	6–8	8–10	10–12
Sample (*n*)	242	79	63	100
Population < 20 μg/L (%)				
Total	3.7	2.5	6.3	3.0
Male	4.8	4.9	8.1	2.1
Female	1.2	0.0	1.6	2.0
Population < 50 μg/L (%)				
Total	7.4	5.1	14.3	5.0
Male	7.9	7.3	16.2	2.1
Female	6.9	2.6	11.5	7.7
Population < 100 μg/L (%)				
Total	25.2	22.8	30.2	24.0
Male	28.6	29.3	35.1	22.9
Female	21.6	15.8	23.1	25.0

**Table 3:** Result of diet questionnaire survey: average days in a week foods were eaten.

Food Type	7 Days (%)	5 Days (%)	3 Days (%)	1 Day (%)	0 (%)	Median (IQR)
Rice	88.0	6.2	4.5	0.8	0.4	7 (7–7)
Noodles	28.9	10.7	35.5	10.3	14.5	3 (2.5–7)
Bread	47.5	10.3	25.2	9.9	7.0	5 (3–7)
Meat	61.4	8.3	17.4	7.9	5.0	7 (3–7)
Vegetables	20.2	10.7	24.0	10.3	34.7	3 (0–5)
Fruits	20.2	9.4	29.5	19.7	21.1	3 (1–5)
Seaweeds	3.3	1.2	4.1	3.7	87.6	0 (0–0)
Agar (jelly)	10.7	3.7	8.3	4.9	72.3	0 (0–1)

IQR = interquartile range.
